# Toxicity Profiling of Bacterial Inclusion Bodies in Human Caco-2 Cells

**DOI:** 10.3389/fbioe.2022.842256

**Published:** 2022-04-29

**Authors:** Irene Barguilla, Ugutz Unzueta, Jose Vicente Carratalá, Olivia Cano-Garrido, Antonio Villaverde, Alba Hernández, Neus Ferrer-Miralles

**Affiliations:** ^1^ Departament de Genètica i de Microbiologia, Universitat Autònoma de Barcelona, Bellaterra, Spain; ^2^ Biomedical Research Institute Sant Pau (IIB Sant Pau), Barcelona, Spain; ^3^ Josep Carreras Leukaemia Research Institute, Barcelona, Spain; ^4^ Networking Center for Biomedical Research in Bioengineering, Biomaterials, and Nanomedicine (CIBER-BBN), Carlos III Institute of Health, Madrid, Spain; ^5^ Institut de Biotecnologia i de Biomedicina, Universitat Autònoma de Barcelona, Bellaterra, Spain; ^6^ Consortium for Biomedical Research in Epidemiology and Public Health (CIBERESP), Carlos III Institute of Health, Madrid, Spain

**Keywords:** inclusion bodies, cytotoxicity, genotoxicity, recombinant protein, caco-2 cells

## Abstract

Bacterial inclusion bodies (IBs) are discrete macromolecular complexes that appear in recombinant prokaryotic cells under stress conditions. These structures are often discarded for biotechnological uses given the difficulty in recovering proteins of interest from them in a soluble form. However, recent approaches have revealed the potential of these protein clusters as biomaterials to promote cell growth and as protein depots for the release of recombinant proteins for biotechnological and biomedical applications. Although these kinds of natural supramolecular complexes have attracted great interest, no comprehensive study of their toxicity in cell cultures has been carried out. In this study, caco-2 cells were exposed to natural IBs, soluble protein-only nanoparticles (NPs), and non-assembled versions of the same protein for comparative purposes. Cytotoxicity, oxidative stress, and genotoxicity were analyzed for all these protein formats. Natural IBs and soluble protein formats demonstrated their safety in eukaryotic cells. No cytotoxicity, genotoxicity, or oxidative stress was detected in caco-2 cells exposed to the protein samples in any of the experimental conditions evaluated, which covered protein concentrations used in previous biological activity assays. These conditions evaluated the activity of protein samples obtained from three prokaryotic hosts [*Escherichia coli* and the endotoxin-free expression systems *Lactococcus lactis* and *ClearColi®* BL21 (DE3)]. Our results demonstrate that natural IBs and soluble protein nanoparticles are non-toxic materials for eukaryotic cells and that this may represent an interesting alternative to the classical unassembled format of recombinant proteins for certain applications in biotechnology and biomedicine.

## Introduction

The global biopharmaceutical market is in expansion and actively seeking novel biomaterials to cover the unmet needs of biomedical and biotechnological applications (Fenton et al., 2018; Puertas-Bartolomé et al., 2021). One type of complex biological product that has invaded the biopharmaceutical market in the recent decades is recombinant proteins, and among them, monoclonal antibodies (mAbs) are blockbuster drugs (Ecker et al., 2015; Sanchez-Garcia et al., 2016). In most of the products approved for clinical uses, the protein of interest is administered by the parenteral route in a soluble form (Ibraheem et al., 2014). Unfortunately, owing to the pharmacokinetic characteristics of antibodies and recombinant proteins in general, a significant portion of the protein does not reach the biological target (Petitcollin et al., 2020). Strategies to cope with this reduced effective dose are being developed, such as the so-called virus-like particles (VLPs) or soluble protein nanoparticles (NPs). These particles are formed by higher-order complexes of several copies of the same recombinant protein, which can mitigate the renal clearance effect but can be concomitantly associated with the formation of protein corona and stimulation of cellular uptake by phagocytic and non-phagocytic cells (Cheng et al., 2015).

In this regard, the formation of protein supramolecular complexes in nature is not an isolated event. In fact, in the recombinant protein area, the presence of protein aggregates has been widely documented in all expression systems tested so far (Kopito, 2000; Rodríguez-Carmona et al., 2015; Rueda et al., 2016; Gifre-Renom et al., 2018; De Marco et al., 2019). At the ultrastructural level, these protein aggregates are stable structures with a basic scaffold formed by intramolecular and intermolecular interactions between beta sheets of the same recombinant protein (De Marco et al., 2019). Embedded in this cage, recombinant protein conformers, corresponding to a mixture of native and non-native protein species, can be released by physical means including complete denaturing processes up to solubilization with mild detergents under non-denaturing conditions (Carratalá et al., 2021). In any case, the evidence supporting the presence of biological activity among the protein subpopulations forming part of these aggregates has completely changed the view of this type of biological material from waste to reusable biocompatible material. Initial studies were directed toward the analysis of enzyme immobilization in repeated cycles for industrial applications (Jäger et al., 2020). Other types of approaches have explored the biocompatible nature of these protein clusters in tissue regeneration approaches (Díez-Gil et al., 2010; Seras-Franzoso et al., 2012; Tatkiewicz et al., 2019) or even as protein depots of releasable active protein with tumor-targeting potential in animal models (Pesarrodona et al., 2019; Céspedes et al., 2020). With all these data taken together, IBs are envisioned as a novel biotechnological platform of nanomaterial based on recombinant proteins. However, despite being a source of biological active protein as well as a nanomaterial with intrinsic biomedical potential, detailed analysis of the toxicity of this type of protein clusters has not been fully explored. The presence of endotoxins in recombinant proteins produced in *E. coli* has been widely described (Magalhães et al., 2007; Mamat et al., 2015). However, this type of compounds can be removed from the purified protein by established methods, which have allowed biopharmaceutical products on the market to be approved, insulin being the paradigmatic example of this expression system (Ferrer-Miralles et al., 2009; Baeshen et al., 2015). In parallel, other prokaryotic expression systems are being developed to produce recombinant proteins lacking these specific toxins. An *E. coli*-derived mutant strain has been modified to inhibit the synthesis route of the lipopolysaccharide of *E. coli* and is now commercially available (*ClearColi*® BL21 (DE3)) (Planesse et al., 2015; Viranaicken et al., 2017). Another strategy is to use Gram-positive microorganisms that do not synthesize endotoxins as Gram-negatives do. An interesting alternative is the Gram-positive *Lactococcus lactis*, which is an accepted food additive by the European Food Safety Authority (EFSA) and considered Generally Recognized as Safe (GRAS) by the U.S. Food and Drug Administration (FDA) (Morello et al., 2008; Gifre-Renom et al., 2018). Therefore, the effect of the presence of endotoxins in prokaryotic systems needs to be addressed and these contaminants need to be eliminated from the final product. In this work, the toxicity of the protein aggregates, known in prokaryotic hosts as inclusion bodies (IBs), was analyzed at the cellular level, taking into consideration the cytotoxicity, genotoxicity, and oxidative stress of the interaction of such protein formats with cultured cells. Two unrelated His-tagged recombinant proteins which can be produced and purified from the soluble and insoluble cell fractions were selected: T22-GFP-H6, a GFP-containing protein that carries an N-terminal domain with the ability to bind to CXCR4 receptor (Unzueta et al., 2017), and IFN-γ-H6, a pleiotropic immunomodulator (Schroder et al., 2004). These analysis were compared with two soluble recombinant proteins formats including NPs (Unzueta et al., 2012; López-Laguna et al., 2019) as well as non-assembled protein counterparts from protein samples obtained from endotoxin-producing cells as well as endotoxin-free prokaryotic expression systems (Supplementary Figure S1).

## Methods

### Bacterial Strains and Plasmids

A synthetic gene encoding the modular protein T22-GFP-H6 was designed in house, produced by Geneart (Thermo Fisher Scientific, Waltham, MA, United States) and subcloned into the expression vector pET22b (Novagen, Merck KGaA, Darmstadt, Germany). T22 is a chemokine receptor (CXCR4) antagonist derived from the polyphemousin II that has an antiparallel β sheet structure stabilized by two disulfide bonds (Murakami et al., 1997). *E. coli* Origami B (Novagen) was used as expression system for the T22-GFP-H6 formats as shown in Table 1 (Protein NPs and IBs). In this *E. coli* strain, the thioredoxin reductase (*trxB*) and glutathione reductase (*gor*) genes are deleted, facilitating disulfide bond formation (Xu et al., 2008). A synthetic gene encoding IFN-γ-H6 was designed in house and produced by Geneart (Thermo Fisher Scientific) as a codon optimized version for *Lactococcus lactis* host and subcloned into pETDuet (Novagen) for *E. coli* expression system and pNZ8148 plasmid (Cm®, MoBiTec GmbH, Göttingen, Germany) for *L. lactis* expression system. *ClearColi*® BL21 (DE3) strain (Lucigen, Middleton, WI, United States) was used as expression system for IFN-γ-H6 protein formats (unassembled protein format and IBs) in an *E. coli* endotoxin-free strain (Table 1). IFN-γ-H6 was also produced in *Lactococcus lactis* expression strain NZ9000 (NICE®, Boca Scientific, Inc., Dedham, MA, United States) (Mireau 2005 AMB) in the same protein formats as in *ClearColi*® (Table 1).

**TABLE 1:** Detailed information of the protein name, cell fraction origin, protein format, and expression system of the recombinant proteins used in the study.

**Sample #**	**Protein name/MW**	**Cell fraction**	**Protein format**	**Expression vector**	**Expression system**
1	IFN-γ-H6 / 18.02	ICF	IBs	pETDuet	*Clearcoli®*
2	IFN-γ-H6 / 18.02	SCF	unassembled	pETDuet	*Clearcoli®*
3	IFN-γ-H6 / 18.02	ICF	IBs	pNZ8148	*L. lactis NZ9000*
4	IFN-γ-H6 / 18.02	SCF	unassembled	pNZ8148	*L. lactis NZ9000*
5	T22-GFP-H6 / 30.7	ICF	IBs	pET22b	*E. coli* Origami B
6	T22-GFP-H6 / 30.7	SCF	Protein NPs	pET22b	*E. coli* Origami B

Each protein was analyzed in two different formats as indicated. ICF, insoluble cell fraction; SCF, soluble cell fraction. Three protein formats were obtained: IBs, inclusion bodies; protein NPs, protein nanoparticles and unassembled soluble protein. MW, molecular weight in kDa.

### Soluble Protein Production and Purification of Soluble Protein Formats

pET22b-T22-GFP-H6 plasmid was transformed into *E. coli* Origami B (Novagen) by heat shock at 42°C and maintained in lysogenic broth (LB) supplemented with 100 μg/ml ampicillin. Recombinant protein was produced in the same medium O/N at 20°C upon induction with 0.1 mmol/L isopropyl- β-d-1-thiogalactopyranoside (IPTG; Sigma–Aldrich, Sant Louis, MO, United States). Cells were then harvested by centrifugation (10 min at 5,000 *g*). T22-GFP-H6 was purified from the soluble cell fraction by affinity chromatography (Supplementary Method). Protein purity was determined by SDS-PAGE gel electrophoresis and subsequent protein immunodetection by Western blot using an anti-His monoclonal antibody (Santa Cruz Biotechnology, Dallas, TX, United States; ref: Sc-57598). Protein integrity was then verified by MALDI-TOF mass spectrophotometry in a linear mode (20–80 kDa). For that, 5 µl of protein sample was first dialyzed against milli-Q H2O in a Millipore membrane for 20min and loaded then onto a MALDI plate with Siapinic Acid in sandwich mode. Final protein concentration was determined by Bradford assay. Formation of protein NPs (generated by the self-assembling of monomeric building blocks of recombinant protein) was determined by dynamic light scanning (DLS) analysis in a Zetasizer Nano ZS (Malvern panalytical, Malvern, United Kingdom). The factors involved in the formation of protein NPs from soluble recombinant proteins have been previously described and seem to be related to the cationic nature of the peptide located at the N-terminal end of the protein and the C-terminal His-tag (Unzueta et al., 2012; Serna et al., 2016).

Cultures of ClearColi® BL21 (DE3) cells transformed with the plasmid pETDuet-IFN-γ-H6 were incubated in a shake flask at 37°C and 250 rpm in LB medium supplemented with 100 μg/ml ampicillin. Protein expression was induced in the same medium by adding 1 mmol/L isopropyl-β-d-thiogalactopyranoside (IPTG). The cultures were then incubated at 20°C and 250 rpm O/N for protein production. Cells were harvested by centrifugation (10 min at 5,000 *g*). IFN-γ-H6 was purified from the soluble cell fraction by affinity chromatography (Supplementary Method). The final protein concentration was determined by Bradford assay, yielding 0.80 mg/ml.

pNZ8148 plasmid (Cm^R^, NICE) was transformed into competent *L. lactis* NZ9000 bacteria, as described elsewhere (Cano-Garrido et al., 2016). Transformed *L. lactis* cells with pNZ8148-IFN-γ-H6 were grown in M17 medium supplemented with 0.5% glucose at 30°C without agitation. Antibiotics were used for plasmid selection such as chloramphenicol (5 μg/ml) and erythromycin (2.5 μg/ml). Protein production was induced in the same medium by adding 500 ng/ml nisin (Sigma–Aldrich). After induction, cultures were grown for 3 h. Bacteria were harvested at 5,000 *g* for 5 min at 4°C and washed twice with PBS. IFN-γ-H6 was purified from the soluble cell fraction by affinity chromatography (Supplementary Method). Protein purity was determined by SDS-PAGE gel electrophoresis and final protein concentration was determined by Bradford assay.

Different protein formats of T22-GFP-H6 and IFN-γ-H6 obtained from the soluble cells fractions of the expression hosts are represented in Supplementary Figure S1.

### Insoluble Protein Production and Purification of IBs

pET22b-T22-GFP-H6 plasmid was transformed into *E. coli* Origami B (Novagen) by heat shock at 42°C and maintained in LB supplememted with 100 μg/ml ampicillin. Recombinant protein was produced in the same medium for 3 h at 37°C upon induction with 1 mmol/L isopropyl- β-d-1-thiogalactopyranoside (IPTG). Cells from 20 ml of culture were then harvested by centrifugation (5 min at 5,000 *g*). Purification of IBs of T22-GFP-H6 was performed following protocol described in Supplementary Method. Protein purity was determined by SDS-PAGE gel electrophoresis and protein concentration was determined by western blot using an anti-His monoclonal antibody (Genescript # A00186) with a calibration curve of quantified soluble recombinant GFP-H6.

pETDuet-IFN-γ-H6 plasmid was transformed into ClearColi® BL21 (DE3) cells and grown as described for the soluble protein version. IB production was induced by adding 1 mmol/L isopropyl-β-D thiogalactopyranoside (IPTG) to ClearColi® BL21 (DE3) cultures grown in LB supplemented with 100 μg/ml ampicillin. After induction, the cultures were grown for 5 h at 37°C. Cells were harvested by centrifugation (10 min at 5,000 g). Purification of IBs of IFN-γ-H6 was performed following protocol described in Supplementary Method **.** The recombinant protein yield was estimated by comparison with a standard curve of known amounts of purified rBoIFN-γ protein quantified by the Bradford assay. Quantification was performed with Image Lab software (Bio-Rad).

pNZ8148-IFN-γ-H6 was transformed into *L. lactis* cells and cells were grown as described in the production of soluble IFN-γ-H6 protein version in this prokaryotic host. IB production was induced by adding 500 ng/ml nisin (Sigma–Aldrich). After induction, cultures were grown for 5 h. Final OD_550 nm_ was 3.02. Bacteria were harvested at 5,000 *g* for 5min at 4°C and washed twice with PBS. Purification of IBs of IFN-γ-H6 was performed following protocol described in Supplementary Method. Protein purity was determined by SDS-PAGE gel electrophoresis, and protein concentration was determined by western blot using an anti-His monoclonal antibody (Genescript # A00186) with a calibration curve of quantified soluble recombinant GFP-H6.

Schematic representation of purification of IBs of T22-GFP-H6 and IFN-γ-H6 obtained from the insoluble cells fractions of the expression hosts are showed in Supplementary Figure S1.

### Cell Culture Conditions

Caco-2 cells were grown in Dulbecco’s modified Eagle’s medium (DMEM) free of sodium pyruvate (Biowest, Nuaillé, France), supplemented with 10% fetal bovine serum, 1% non-essential amino-acids (Biowest) and 2.5 μg/ml Plasmocin (InvivoGen, San Diego, CA, United States) in a humidified atmosphere of 5% CO2 and 95% air at 37°C. This cell line was used as it provided low or absent expression of CXCR4 to which T22 peptide acted as an antagonist (Tamamura et al., 2006; Rubie et al., 2011; Stuckel et al., 2020).

### Cell Viability Assay

Cell viability was determined by the Beckman counter method using a ZTM Series Coulter-Counter (Beckman Coulter Inc. Bea, CA, United States). Briefly, 150,000 cells per well were seeded on 12-well plates. The next day, the cells were exposed to increasing concentrations of the different protein formats of T22-GFP-H6 (ranging from 4 × 10^3^ to 70 × 10^3^ ng/mL, or what is the same, 0,14 to 2.3 μmol/L) and IFN-γ-H6 (ranging from 18 to 180 ng/ml, or the same, 1–10 μmol/L). After 24 h, cells were counted, and survival curves were represented.

### Cell Death, Necrosis, and Apoptosis Assay

The proportion of live, apoptotic, and necrotic cells was assessed with Annexin V FLUOS staining kit (Sigma–Aldrich) following the manufacturer’s protocol. Thus, cells were seeded at a density of 150,000 cells per well in 12-well plates and the following day, they were treated with two different concentrations of each protein: 18 × 10^3^ ng/ml and 70 × 10^3^ ng/ml T22-GFP-H6, and 72 and 180 ng/ml IFN-γ-H6. After 24 h of exposure, the supernatant was collected in a 15 ml tube. Adherent cells were trypsinized and collected in the same tube as the supernatant for each condition. The mix was centrifuged and washed once with PSB 1X. The pellet was resuspended in 100 µl of incubation buffer with 2 µl of Annexin V and 2 µl of propidium iodide (PI). Unstained cells and resuspended cell mixture were used as compensation controls to improve the gating strategy. The cells were incubated for 15 min at room temperature and in the dark for staining before adding 100 µl more of incubation buffer per sample. Then, samples were analyzed by BD FACSCanto Flow Cytometer (BD Biosciences, Franklin Lakes, NJ, United States). More than 10,000 events per sample were analyzed. Among these events, the PI-negative and Annexin V-negative cells represent the living population, PI-negative and Annexin V-positive cells are considered apoptotic and cells positive for both PI and Annexin V are considered necrotic. Cells exposed for 30 min to 100 mM H_2_O_2_ were used as positive controls.

### Comet Assay

The alkaline single cell gel electrophoresis (Comet) assay is a powerful technique to detect directly both oxidative stress and its effects inducing DNA strand breaks at the level of individual cells. Here it was used to evaluate the genotoxic and oxidative DNA damage for caco-2 cells exposed to the different protein forms, using formamidopyrimidine DNA glycosylase (FPG) as previously described (Bach et al., 2014). Gelbond® films (GF) were used for this assay. Cells were exposed for 24 h to high concentrations of the different recombinant proteins and forms: 4, 8, 18, 36, and 70 μg/ml. The next day, the cells were trypsinized, collected, centrifuged, and resuspended in cold PBS 1X at a concentration of 1 × 10^6^ cells/mL. In the following step, cells were mixed with 0.75% low-melting-point agarose at 37°C and three drops of 7 μl each were placed in the GF. Two GF with identical samples were processed simultaneously in each experiment. Then, a lysis step was performed overnight by immersing the GF in ice-cold lysis buffer at 4°C (2.5 mol/L NaCl, 0.1 mol/L Na_2_EDTA, 0.1 mol/L Tris base, 1% Triton X-100, 1% lauroyl sarcosinate, 10% DMSO), at pH 10. The following day, the GF replicates were gently washed twice (1 × 5 min, 1 × 50 min) in enzyme buffer at 4°C and pH 8 (10 mmol/L HEPES, 0.1 mol/L KCl, 0.5 mm l/L EDTA, 0.2 mg/ml BSA) and then, they were incubated for 30 min at 37°C in enzyme buffer (negative control) or FPG-containing enzyme buffer. Later, the GF were washed with electrophoresis buffer and placed in a horizontal electrophoresis tank for 35 min to allow the DNA unwinding 0.3 ml/L NaOH and 1 mmol/L Na_2_EDTA pH 13.2 before the electrophoresis, which was carried out for 20 min at 0.8 V/cm and 300 mA at 4°C. Then, GF were rinsed with cold PBS for 15 min and fixed in absolute ethanol for 2 h before air-drying it overnight at room temperature. A staining step was performed by incubating the GF for 20 min with SYBR Gold 1/10,000 in TE buffer (10 mmol/L Tris, 1 mmol/L EDTA pH 7.5). Finally, gels were mounted, visualized for comets using an epifluorescent microscope at ×20 magnification, and analyzed with the Komet 5.5 Image analysis system (Kinetic Imaging Ltd., Liverpool, United Kingdom). The levels of DNA damage were evaluated according to the percentage of DNA in the tail of the cells. One hundred randomly selected comet images were analyzed per sample. Cells exposed to the DNA damaging agent methyl methanesulfonate (MMS,Sigma–Aldrich) at 200 μmol/L for 1 h at 37°C were used as controls.

### Statistics

Analysis of variance followed by Dunnett’s multiple comparison test was performed appropriately to compare the effect of the exposure to the different recombinant proteins. A two-sided *p* < 0.05 was considered statistically significant in all cases.

## Results

### Analysis of Purified Recombinant Proteins

Gel electrophoresis of T22-GFP-H6 and IFN-γ-H6 showed the expected protein bands (Supplementary Figure S2). In the soluble cell fraction (Supplementary Figure S2A) the main protein band corresponded to the recombinant protein showing a purity >90%. Some other accompanying protein bands of higher molecular weight corresponded to a small proportion of quaternary structures. In fact, dimerization of GFP (Wiedenmann et al., 2000) and IFN-γ (Fensterl and Sen, 2009) has been widely described and observed even under denaturing conditions of the SDS-PAGE electrophoresis. The presence of protein NPs in the T22-GFP-H6 sample was determined by DLS (Supplementary Figure S4) as previously described (Céspedes et al., 2016). Protein identification in the insoluble cell fraction was performed by immunodetection (Supplementary Figure S2B). In each sample, the protein band compatible with the corresponding expected molecular weight could be detected although analysis on polyacrylamide gels showed variable purity (Supplementary Figure S3). In fact, the relative presence of the recombinant protein in such samples depends on the protein itself and the experimental conditions set during gene expression with a wide range of purity between experiments for different proteins (Peternel et al., 2008; Castellanos-Mendoza et al., 2014; Carratalá et al., 2020b). The presence of associated proteins as chaperones and other cellular components in IBs have been widely described (Ventura and Villaverde, 2006).

### Toxicological Assessment of the Recombinant Proteins

**FIGURE 1:**
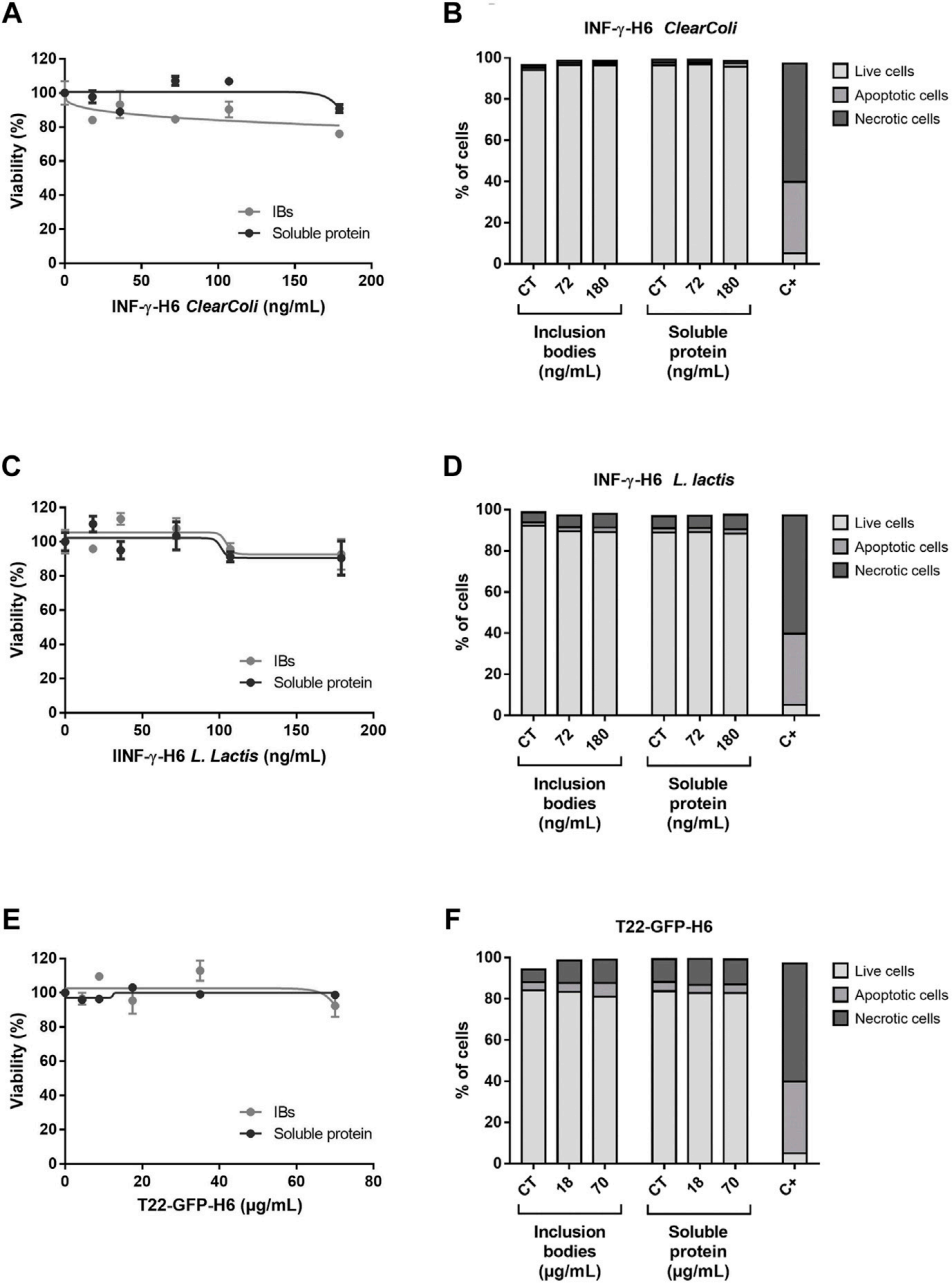
Cytotoxicity of recombinant proteins. **(A,C,E)** Cell viability of caco-2 exposed for 24 h to increasing doses of INF-γ-H6 from *ClearColi®,* INF-γ-H6 from *L. lactis* and T22-GFP-H6, respectively. Data are presented as number of exposed cells relative to the non-exposed controls ±SEM. **(B,D,F)** Percentage of live, apoptotic, and necrotic cells in the non-exposed controls, and cells exposed for 24 h to increasing doses of INF-γ-H6 from *ClearColi®,* INF-γ-H6 from *L. lactis* and T22-GFP-H6, respectively. The positive control corresponds to the percentage of live, apoptotic, and necrotic cells after 30 min of exposure to H_2_O_2_. The data determined by Annexin V staining are presented as mean % ± SEM.

**FIGURE 2:**
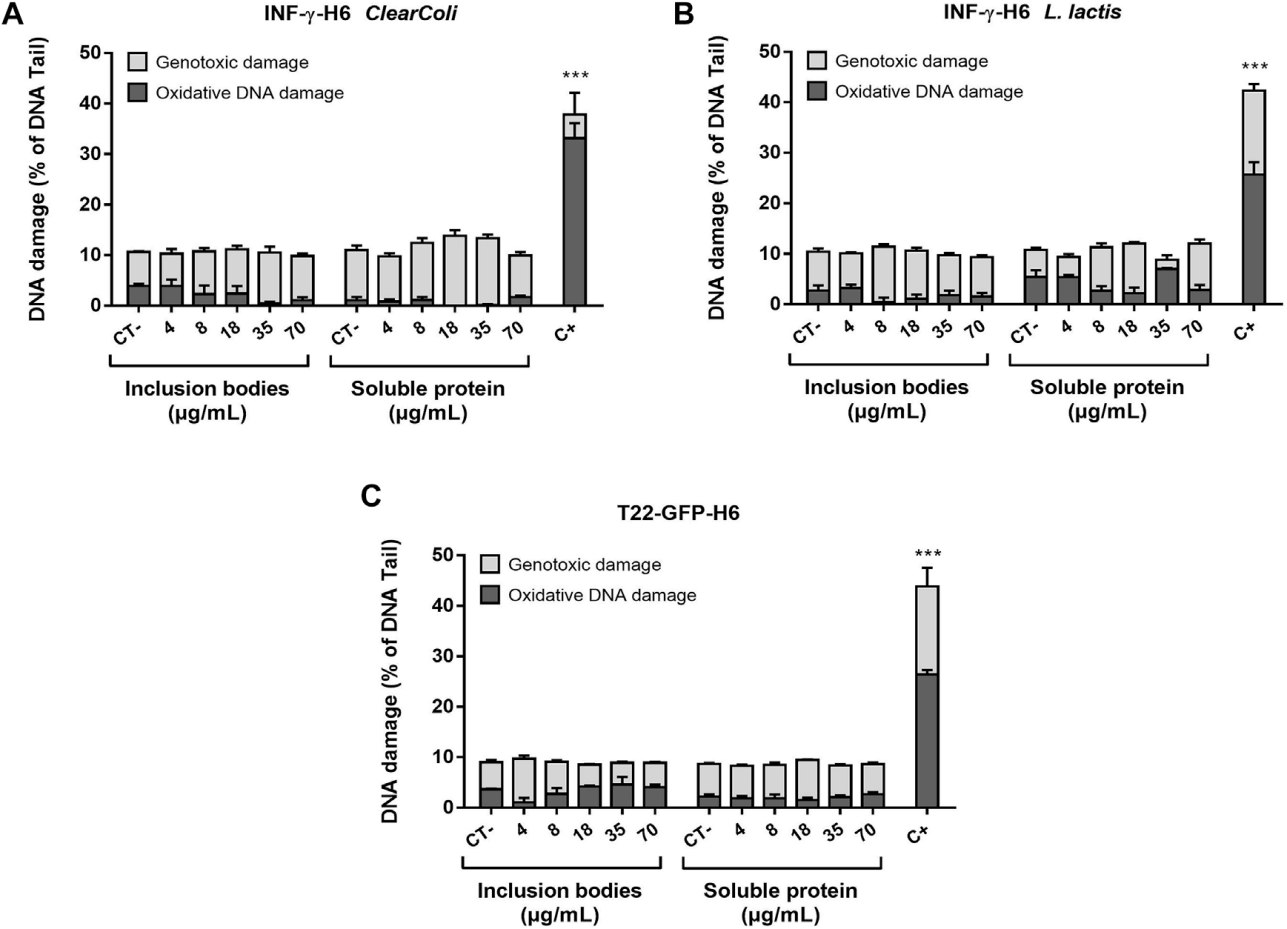
Genotoxic and oxidative DNA damage induced by the recombinant proteins. DNA damage level on cells exposed to increasing doses of **(A)** INF-γ-H6 from *ClearColi®*, **(B)** INF-γ-H6 from *L. lactis*, and **(C)** T22-GFP-H6 after 24 h. 1 h exposure to MMS (200 μmol/L) was used as a positive control. Data are presented as mean % of DNA quantified in the comet tail ±SEM.

The analysis of diverse endpoints linked to the toxicity potential *in vitro* showed that neither T22-GFP-H6 nor IFN-γ-H6 purified from *ClearColi®* or *L. lactis* were toxic in any of the protein forms assessed (natural IBs or soluble protein formats). Caco-2 cells had a cell survival rate generally over 80% when exposed to concentrations within the standard range, and no significant differences were found between exposed cells and non-exposed controls (Figures 1A,C,E). This was further confirmed by the results from the PI and Annexin V staining followed by flow cytometry analysis, where living cells were again found to be above 80% for all concentrations tested (Figures 1B,D,F), and in any case, these percentages were significantly different from the non-exposed controls. The proportion of apoptotic and necrotic cells in the culture was characterized for an intermediate and a high concentration of exposure (72 and 180 ng/ml for IFN-γ-H6; and 18 × 10^3^ and 70 × 10^3^ ng/mL of T22-GFP-H6), confirming the results of the cell viability assays (Figures 1B,D,F). Approximately 2% of the cell population were apoptotic cells in those exposed to IFN-γ-H6, while around 4% of the cells were apoptotic after exposure to T22-GFP-H6. Necrotic cells represented 1% of the caco-2 cells exposed to IFN-γ-H6 from *ClearColi*®, 6% of the cells exposed to IFN-γ-H6 from *L. Lactis*, and 10% of the cells exposed to T22-GFP-H6. Nonetheless, none of these percentages were significantly different from the proportions observed in non-exposed cells. In line with the above, the analysis of oxidized DNA bases by the comet assay with the inclusion of FPG enzyme also points out toward the absence of induced oxidative stress, as oxidative DNA damage did not exceed 10% for any of the conditions tested (Figure 2). Importantly, the analysis of DNA damage in individual cells by the Comet assay revealed no genotoxic effects of the recombinant proteins despite the increment in the dose of exposure. Genotoxic DNA damage levels did not differ from those found in the control cells, with values no higher than 12% (Figure 2). There were no significant differences in the levels of DNA damage when comparing T22-GFP-H6, IFN-γ-H6 from *ClearColi®*, and IFN-γ-H6 from *L. lactis*, nor between IBs or the soluble form of the different proteins.

## Discussion

Recombinant proteins play an essential role in diagnosis and therapy. The *E. coli* expression system is one of the most established hosts, generating more than 20% of biopharmaceuticals on the market (Baeshen et al., 2014). However, although the steady increase in mAbs approvals favors the trend toward mammalian over non-mammalian expression hosts (Walsh, 2018), in cancer therapies, prokaryotic host-derived products account for more than 60% of marketed products (Sanchez-Garcia et al., 2016).

One of the disadvantages of recombinant protein production in *E. coli* is the formation of IBs, which have been classically regarded as undesirable by-products. However, the detailed characterization of this type of protein aggregate has revealed the presence of protein molecules with native-like structure, which can be recovered by diffusion from the scaffold structure of the IBs under non-denaturing conditions (Peternel et al., 2008; Singh et al., 2015; Gifre-Renom et al., 2018). In addition, IBs have been demonstrated to facilitate cell growth of cultured cells on decorated surfaces and 3D scaffolds as well as to release recombinant protein in a sustained manner when administered subcutaneously (Seras-Franzoso et al., 2012; Seras-Franzoso et al., 2013; Céspedes et al., 2020). These properties have raised interest in this biomaterial for biomedical applications (De Marco et al., 2019). For this reason, toxicological assessment of IBs needs to be addressed.

In this study, IBs were obtained from three different prokaryotic expression hosts in order to discriminate the putative toxicity of IBs obtained from *E. coli*, which produces endotoxins; from an *E. coli* strain (*ClearColi*®) that produces a genetically modified version of lipopolysaccharide (LPS), which does not induce endotoxin response in mammalian cells; and from *L. lactis*, an LPS-free host (Wakelin et al., 2006; Morello et al., 2008; Mamat et al., 2015). In all cases, two protein formats were obtained, the soluble protein and the corresponding IBs, for comparison reasons. The results presented here indicate that neither the protein format nor the origin of the protein samples had any effect on the toxicity assays performance on caco-2 cells. The presence of endotoxins in the protein samples obtained from *E. coli* has been widely described for soluble counterparts (Schwarz et al., 2014) and in IB products (Stanek et al., 2019). Even though the LPS contents of the protein samples obtained in this study were not determined, it can be assumed that this product was present at the same level. In the case of proteins obtained from the other two expression systems, the presence of LPS might be residual and only a product of cross-contamination arising from sharing the same laboratory facilities, equipment, and material. In fact, the protein concentrations used in this study mirrored previous experimental approaches used in cell culture assays to assess the biological activity of the recombinant proteins (Serna et al., 2019; Carratalá et al., 2020a; Carratalá et al., 2020c; Álamo et al., 2020). The soluble version and the IBs format of T22-GFP-H6 have been tested for the selective interaction with CXCR4+ cells to target metastatic cells in a variety of cancer types as well as their performance as an antimicrobial agent due to the presence of the T22 peptide (polyphemusin) isolated from the hemocytes of horseshoe crabs (Céspedes et al., 2016; Falgàs et al., 2020a; Falgàs et al., 2020b; Céspedes et al., 2020; Serna et al., 2021). The IFN-γ proteins, in both formats, have been assayed in cultured cells and in animal models to check the immunostimulatory effect of the cytokine in infectious diseases (Carratalá et al., 2020a; Carratalá et al., 2020c). The lack of induction of cytotoxicity, cell death, apoptosis, and necrosis, oxidative stress, and genotoxicity of any of the proteins and the protein formats tested in this study indicates that this type of protein complex may represent an appealing platform of protein-based biomaterials from which to explore their use in the development of novel protein formats in biomedical applications including diagnosis and therapy.

## Data Availability

The raw data that support the findings of this work are available in UAB Digital Repository of Documents at https://ddd.uab.cat/record/257475.
