# Supplementary material for: Toxicity Profiling of Bacterial Inclusion Bodies in Human Caco-2 Cells
Source: Front Bioeng Biotechnol. 2022 Apr 29;10:842256. doi: 10.3389/fbioe.2022.842256 (PMC9099286; doi:10.3389/fbioe.2022.842256)
Supplement: Supplementary file 1 [file DataSheet1.docx]

Supplementary Material

# Supplementary Method

## Purification of Soluble Protein Formats

*E. coli* Origami B cell pellets for the expression of T22-GFP-H6 were resuspended in wash buffer (20 mmol/L Tris-HCl pH 8, 500 mmol/L NaCl, 10 mmol/L Imidazole) supplemented with protease inhibitors (cOmplete EDTA free, Roche, Merck KGaA, Darmstadt, Germany) for cell disruption in a French Press (Thermo FA-078A) (two rounds at 1200 psi). The soluble cell fraction was then recollected by centrifugation (45 min at 15000 g) and charged in a HiTrap Chelating HP column (Cytiva, Marlborough, MA, USA) for an immobilized metal affinity chromatography (IMAC) in an ÄKTA Pure system (Cytiva). Protein elution was achieved by a step gradient profile of elution buffer (20 mmol/L Tris-HCl pH 8, 500 mmol/L NaCl, 500 mmol/L Imidazole). Pure protein fractions were then pooled and dialyzed against sodium carbonate with salt buffer (166 mmol/L NaCO3H, 333 mmol/L NaCl, pH 8).

Cell pellets of ClearColi® BL21 (DE3) cells transformed with the plasmid pETDuet-IFN-γ-H6 were resuspended in wash buffer (20 mmol/L Tris-HCl pH 8, 500 mmol/L NaCl, 10 mmol/L Imidazole) and supplemented with protease inhibitors (cOmplete EDTA free, Roche). Cell lysis was performed by sonication (1 round of 3 min at 10 % amplitude and 5 rounds of 3 minutes at 15 % amplitude; Branson 450 Digital Sonicator). The resultant solution was centrifuged (15,000 *g*, 40 min, 4 °C) to separate cell debris from soluble elements, after that, the soluble cell fraction was filtered by 0.22 μm and kept on ice. Protein purification was carried out by His tag affinity chromatography using ÄKTA Purifier FPLC (Cytiva) and HiTrap Chelating HP 1 mL column (Cytiva). The sample was charged into the column at a flow rate of 1 mL/min, after that, the column was washed with five column volumes and elution was set with a linear gradient of 20 CV at a flow rate of 1 mL/min (Elution buffer: Tris-HCl 20 mmol/L pH 8.0, 500 mmol/L NaCl, 500 mmol/L Imidazole). All fractions were collected and analyzed by TGX technology (Bio‐Rad Laboratories, Inc, Hercules, CA, USA) and western blot using an anti-His monoclonal antibody (Santa Cruz Biotechnology, ref: Sc-57598)). The positive IFN‐γ-containing fractions (13 mL) were eluted in a single peak, pooled, and dialyzed against phosphate‐buffered saline buffer (PBS; 139 mmol/L NaCl, 7.5 mmol/L Na_2_HPO_4_, and 2.5 mmol/L NaH_2_PO_4_; pH 7.4) overnight at 4 °C.

*L. lactis* NZ9000 cell pellets containing IFN-γ-H6 were resuspended in 20 mL of lysis buffer (20 mmol/L Tris-HCl pH 8, 500 mmol/L NaCl, 10 mmol/L Imidazole) and supplemented with protease inhibitors (cOmplete EDTA free, Roche). Bacterial cells were lysed in a French Press at 15,000 PSI and the lysis cycle was repeated three times. Soluble cell fraction was recovered by centrifugation (45 min at 15000 *g*) and charged in a HiTrap Chelating HP column (Cytiva) for an immobilized metal affinity chromatography (IMAC) in an ÄKTA Pure system (Cytiva). Protein elution was achieved by a step gradient profile of elution buffer (20 mmol/L Tris-HCl pH 8, 500 mmol/L NaCl, 500 mmol/L Imidazole). The protein fractions obtained at 15 % and 30 % elution steps were discarded and protein fractions obtained at 100 % elution buffer were pooled and dialysed against PBS.

## Purification of IBs

*E. coli* Origami B cell pellets for the expression of T22-GFP-H6 were resuspended in 20 mL of lysis buffer (50 mmol/L Tris-HCl pH 8.1, 100 mmol/L NaCl, and 1 mmol/L EDTA) and stored at -80 °C. Thawed suspension was supplemented with 0.5 mmol/L phenylmethanesulphonylfluoride (PMSF) and 1 mg/mL lysozyme. The solution was incubated at 37 °C for 2 h and further incubated for 1h at room temperature after the addition of 0.5 % Triton X-100. The mixture was kept on ice and sonicated for 4 to 10 cycles of 10 min at 40 % amplitude under 0.5 s cycles. After sonication, cell viability was determined by seeding 100 µL on LB plates with the corresponding antibiotics, and incubated O/N at 37 °C. 5 µL of Nonidet P40 (NP-40) were added to the mixture and incubated at 4 °C for 1 h. Degradation of contaminating DNA was performed by incubating the lysate for 45 min at 37 °C upon addition of 15 µL of DNase (1mg/mL) and 0.75 mmol/L MgSO_4_. Insoluble fraction containing pure IBs was then recovered by centrifugation (4 °C, 15 min at 15000 *g*), and was washed with 1 mL of lysis buffer supplemented with 0,5 % Triton X-100. Pellets were stored at - 80 °C.

Cell pellets of ClearColi® BL21 (DE3) cells transformed with the plasmid pETDuet-IFN-γ-H6 were resuspended in PBS and disrupted by French press (3 cycles at 1,500 psi). After that, lysozyme (10 μg/mL) (Roche # 10 837 059 001) was added and incubated 2 h at 37 °C 250 rpm and after this it was frozen at ‐80 °C O/N. After thawing the mixture, Triton X‐100 (0.4 mL/100 mL sample) (Roche # 10 789 704 001) was added, and the sample was incubated at RT 1 h under agitation. At this point, bacterial contamination was tested by inoculating 100 μL of the mixture on LB plates. Freeze/thaw cycles were repeated until non-viable cells were observed on LB plates. Then, Nonidet P‐40 (25 μL/100 mL sample) (Roche # 11 754 599 001) was added, and the mixture was incubated 1 h under agitation at 4 °C. Following this, MgSO_4_ (60 μL/100 mL sample) and DNAse I (60 μL/100 mL sample) (Roche # 10 104 159 001) were added and the sample was incubated 1 h under agitation at 37 °C. Finally, the pellet was resuspended in lysis buffer + Triton X‐100 (5 mL/100 mL initial sample) and harvested at 15,000 *g* for 15 min at 4 °C. Another freeze/thaw cycle was done, IBs were harvested at 15,000 *g* for 15 min at 4 °C, the supernatant was discarded, and the IBs were diluted and resuspended 1:10 in PBS. The protocol was performed under sterile conditions.

*L. lactis* NZ9000 cell pellets containing IFN-γ-H6 and corresponding to 50 mL of medium were resuspended in 30 mL of filtered PBS and stored at -80 °C for 2 h. Cell suspension was thawed on ice and at least three freeze/thaw cycles were performed. Bacterial cells were lysed in a French Press at 15,000 PSI and the lysis cycle was repeated three times. Remaining viable cells were monitored by incubating 100 µL of the lysate on M17 plates O/N at 30 °C. Harvest IBs at 10,000 *g* for 5 min at 4 °C. Resuspend in 30 mL PBS with lysozyme at 0.01mg/mL and incubate the mixture for 2 h at 37 °C. After one freeze/thaw cycle, 120 µL of triton X-100 was added, and the solution was incubated for 1h at room temperature. Following another freeze/thaw cycle, 7.5 mL of NP-40 were added, and the solution was incubated for 1 h at 4 °C. Degradation of contaminating DNA was performed by incubating the lysate for 1 h at 37 °C upon addition of 23 µL of DNase (1mg/mL) and 0.75 mmol/L MgSO4. Purified IBs were harvested by centrifugation at 15,000 g for 30 min at 4 °C and stored at -80 °C. Aliquots of IBs corresponding to 20 mL of induced culture were separated in Eppendorf tubes.

# Supplementary Figures

## Supplementary Figure 1


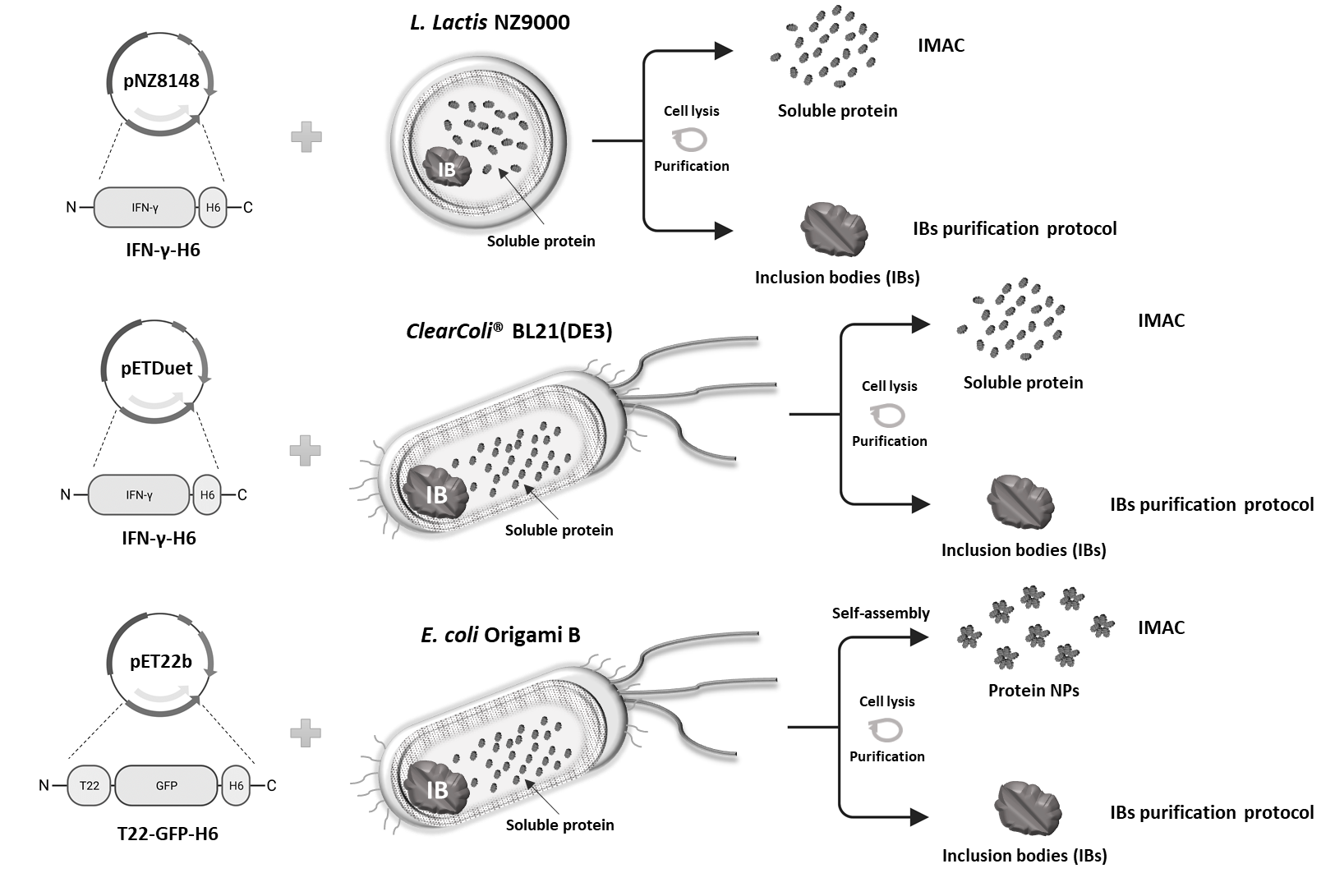


**Supplementary Figure 1 |** Schematic representation of protein formats obtained from a prokaryotic expression system. The presence of recombinant protein accumulated in the cytosol is shown, other cell components are not shown for simplicity. After cell lysis and centrifugation, two separate cell fractions are obtained (soluble and insoluble cell fractions). From the soluble cell fraction the purified recombinant protein is usually recovered as unassembled species. However, in the design of the recombinant protein, specific architectonic domains can be added to enhance the formation of higher-order complexes (protein nanoparticles-NPs). IBs are obtained from the insoluble cell fraction as described in the Method section. IMAC: Immobilized metal affinity chromatography

## Supplementary Figure 2


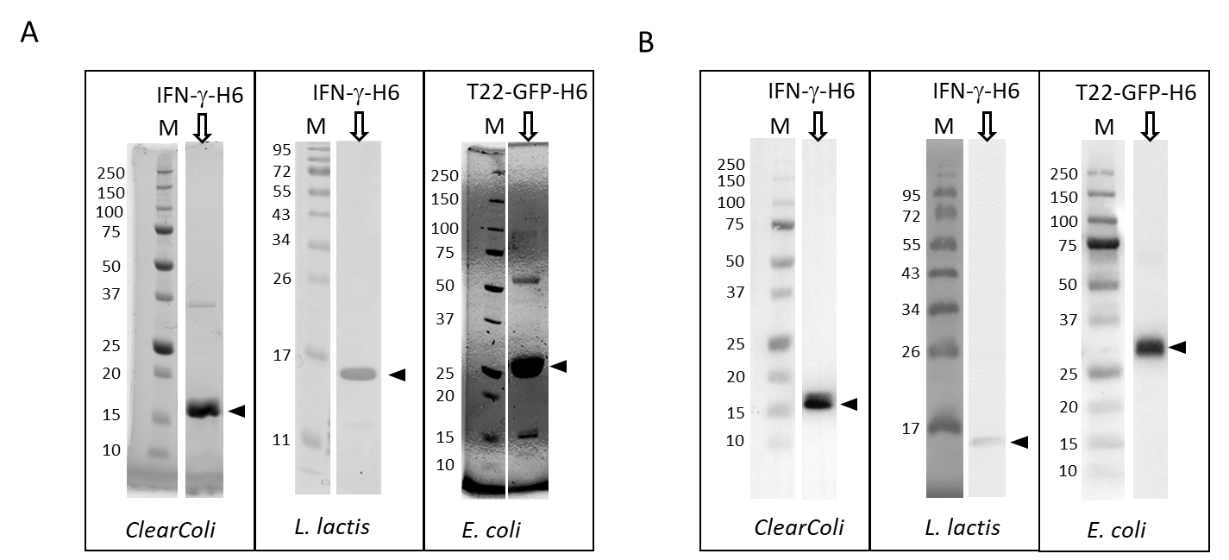


**Supplementary Figure 2 |** Purified recombinant proteins. **(A)** SDS-PAGE of proteins purified from the soluble cell fraction of the indicated expression host. **(B)** Western blot detection of recombinant proteins in purified IBs of the indicated expression host by. Numbers on the left indicate the molecular weight in kDa of the ladder marker. On the right, arrows indicate the position of the recombinant proteins. M indicates the molecular marker line. The theoretical expected molecular weight was as it follows: T22-GFP-H6 (30.7 kDa) and IFN-γ-H6 (18.02 kDa).

## Supplementary Figure 3

**Supplementary Figure 3** | SDS-PAGE gels loaded with purified IBs from the insoluble cell fraction of the indicated expression host. Numbers on the left indicate the molecular masses in kDa of the ladder marker. On the right, arrows indicate the position of the recombinant proteins. M indicates the molecular marker line. T22-GFP-H6 (30.7 kDa) and IFN-g-H6 (18.02 kDa).

## Supplementary Figure 4


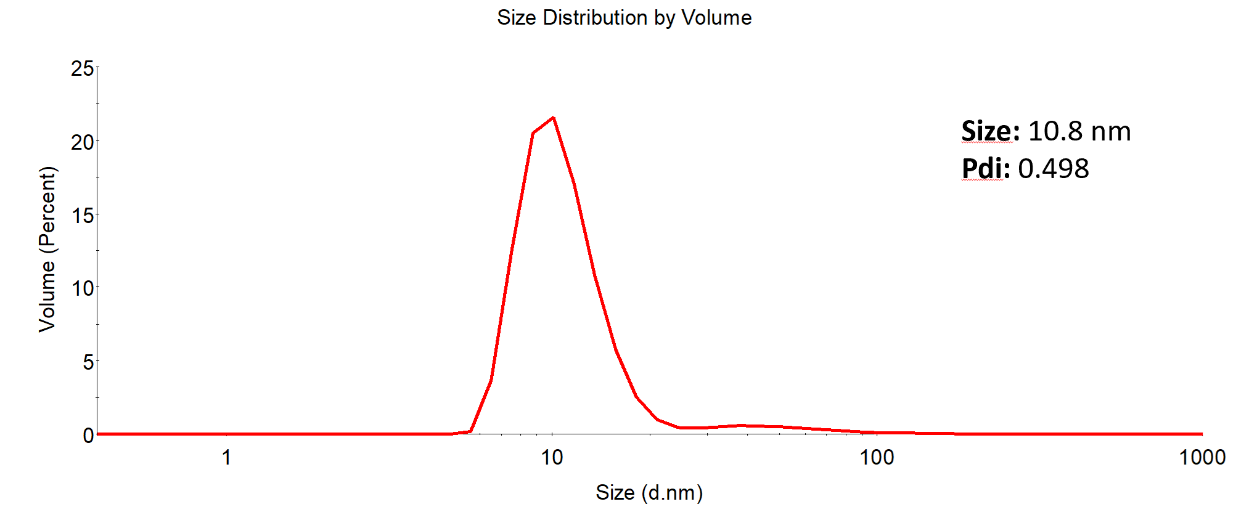


**Supplementary Figure 4 |** Size distribution of soluble of T22-GFP-H6 (at 1 mg/mL) in carbonate buffer analyzed by dynamic light scattering (DLS). Pdi: polydispersity index.
